# The Use of Polymethylmethacrylate Cement in Percutaneous Vertebroplasty Versus Conservative Management: How to Treat Osteoporotic Vertebral Compression Fractures

**DOI:** 10.3390/medicina61112004

**Published:** 2025-11-09

**Authors:** Corrado Ciatti, Chiara Asti, Pietro Maniscalco, Michelangelo Rinaldi, Gianfranco Pirellas, Gianfilippo Caggiari, Francesco Pisanu, Angelino Sanna, Carlo Doria

**Affiliations:** 1 Department of Medicine and Surgery, University of Parma, 43126 Parma, Italy; 2 Department of Orthopedics and Traumatology, Guglielmo da Saliceto Hospital, Via Taverna 49, 29121 Piacenza, Italy; c.asti@studenti.uniss.it; 3 Orthopaedic and Traumatology Department, Università Degli Studi di Sassari, 07100 Sassari, Italy; michelangelo.rinaldi92@gmail.com (M.R.); gianfrancopirellas@gmail.com (G.P.); gianfilippocaggiari@gmail.com (G.C.); pisanuf@gmail.com (F.P.); angelino.sanna@aousassari.it (A.S.); cdoria@uniss.it (C.D.); 4 Department of Medicine and Aging Sciences, University of Chieti-Pescara, 66100 Chieti, Italy; pietro.maniscalco@unipr.it

**Keywords:** osteoporotic vertebral compression fracture, percutaneous vertebroplasty, conservative management, polymethylmethacrylate, bone fragility, minimally invasive spine surgery

## Abstract

*Background and Objectives*: Osteoporotic vertebral compression fractures (OVCFs) are a major cause of morbidity, disability, and loss of independence in the elderly population. The optimal management of these fractures remains debated, especially regarding the balance between conservative treatment and minimally invasive surgical techniques such as percutaneous vertebroplasty (VP). This study aimed to compare clinical and radiological outcomes of VP and conservative management in patients with acute OVCFs. *Materials and methods*: A retrospective observational cohort study was conducted on 120 patients with acute OVCFs treated either conservatively or through percutaneous VP using polymethylmethacrylate (PMMA) cement. Clinical outcomes were assessed using the Visual Analogue Scale (VAS) for pain, Roland–Morris Disability Questionnaire (RMDQ), and Oswestry Disability Index (ODI). Evaluations were performed at baseline and at 1, 3, 6, and 12 months post-treatment. Radiological follow-up assessed fracture healing and new vertebral fractures. *Results*: Patients treated with VP experienced significantly faster pain relief and functional improvement than those managed conservatively, with marked differences in VAS, RMDQ, and ODI scores within the first month (*p* < 0.01). By 12 months, outcomes converged between groups, with comparable pain and functional levels. No major complications were reported; cement leakage was asymptomatic, and no neurological or systemic adverse events occurred. Radiological healing was satisfactory in both groups, without increased risk of adjacent fractures in the VP group. *Conclusions*: Percutaneous vertebroplasty resulted in faster short-term improvement compared with conservative treatment, while functional outcomes converged over time. The retrospective, non-randomized design limits causal inference.

## 1. Introduction

Osteoporotic vertebral compression fractures (OVCFs) represent one of the most frequent causes of morbidity in the aging population [1]. These fractures occur in patients with osteoporosis, a systemic skeletal disease characterized by reduced bone mass and deterioration of bone microarchitecture, which leads to increased fragility [1,2]. OVCFs are typically the consequence of low-energy trauma or may even occur spontaneously [3,4,5,6]. With progressive demographic aging, the incidence of these fractures is steadily increasing. In 2010, approximately 560,000 new cases were estimated in the European Union, and it is well established that 65 to 75% of OVCFs remain clinically silent [1,3,7].

The pathophysiology of OVCFs involves not only decreased bone mineral density but also impaired bone quality, changes in trabecular microarchitecture, and alterations in the mechanical loading of the spine. As a result, even minor trauma can lead to vertebral collapse, progressive kyphotic deformity, and chronic pain.

In elderly patients, these deformities are associated with reduced pulmonary function, impaired mobility, and increased risk of subsequent fractures, ultimately compromising independence and quality of life. The psychological impact of chronic back pain and loss of autonomy further contributes to the overall disease burden.

Treatment options can be conservative or surgical, and in both cases, the objectives are the relief of pain, restoration of mobility, and prevention of further fractures [4]. The choice between conservative treatment and vertebral augmentation procedures, particularly percutaneous vertebroplasty (VP), remains a matter of debate. Conservative treatment, based on bed rest, analgesic therapy, and orthotic support, has long been considered the gold standard. Nevertheless, surgical procedures such as VP or balloon kyphoplasty have been shown to achieve more rapid pain relief and functional improvement in many cases [8,9]. Over the past two decades, VP has evolved from an experimental technique to a widely accepted minimally invasive procedure, especially in cases of acute, painful fractures refractory to medical therapy. The injection of polymethylmethacrylate (PMMA) cement into the fractured vertebral body provides immediate mechanical stabilization and pain reduction by eliminating micromovements between trabecular fragments and by thermally denervating local nociceptors.

Beyond the immediate clinical implications, OVCFs are increasingly recognized as a sentinel event in the natural history of osteoporosis, often marking the transition from a subclinical to a clinically overt phase of bone fragility [1,2]. Patients experiencing one vertebral fracture have a markedly increased risk—up to fivefold—of sustaining subsequent fractures within the following year [3,4]. This “fracture cascade” effect highlights the need for early intervention strategies that combine mechanical stabilization, pharmacologic treatment, and structured rehabilitation to prevent recurrence and maintain postural alignment [5,6].

The diagnosis of acute OVCFs has also evolved significantly with the introduction of advanced imaging modalities such as Short-Tau Inversion-Recovery Magnetic Resonance Imaging (STIR-MRI) and bone scintigraphy, which allow differentiation between acute and chronic lesions [7,8]. Early and accurate identification of fracture acuity is critical for guiding treatment selection and timing, avoiding unnecessary procedures in chronic cases while ensuring timely intervention in symptomatic acute fractures.

Furthermore, recent attention has been directed toward the biological response of osteoporotic bone to mechanical stabilization. Several experimental models have shown that internal stabilization with polymethylmethacrylate cement may influence local microstrain distribution and stimulate secondary remodeling processes at the bone–cement interface [9,10,11]. Understanding these biomechanical and histological mechanisms is essential to interpret the clinical efficacy and long-term safety of vertebral augmentation.

Finally, the management of OVCFs should not be considered merely a technical issue but a component of a broader multidisciplinary approach. Integration of orthopedic, radiologic, and metabolic expertise, combined with early initiation of anti-osteoporotic therapy, is increasingly regarded as the cornerstone of comprehensive fracture care [12,13,14].

The socioeconomic burden of OVCFs is also considerable. In 2011 fragility fractures were estimated to cost approximately GBP 2 billion in the United Kingdom, while in the United States, more than one billion dollars are spent annually to manage OVCFs [4,5]. The increasing prevalence of osteoporosis worldwide, driven by aging populations, sedentary lifestyles, and nutritional factors, underscores the urgency of identifying effective and cost-efficient management strategies. Optimizing treatment strategies is therefore essential not only for patient outcomes but also for healthcare sustainability.

The present study aimed to compare the clinical and radiological outcomes of percutaneous vertebroplasty and conservative management in patients with acute osteoporotic vertebral compression fractures. We hypothesized that vertebroplasty would provide faster pain relief and functional improvement during early follow-up, while long-term outcomes would converge between the two treatments.

## 2. Materials and Methods

This was a single-center, retrospective observational cohort study conducted at the Orthopedic Clinic of the University Hospital of Sassari. All consecutive patients admitted for acute OVCFs during the study period were screened by review of medical records and imaging. Patients received either conservative management or VP according to clinical judgment and patient preference. Data collection and follow-up were partially affected by the COVID-19 pandemic, which temporarily limited access to outpatient services [10]. Consequently, some patients could not complete all scheduled follow-up visits and were excluded from the final analysis.

Inclusion criteria were carefully defined to ensure homogeneity of the study cohort. Patients were required to be between 50 and 89 years of age, to have a confirmed diagnosis of primary osteoporosis based on densitometric criteria, and to have experienced an acute vertebral compression fracture. Only those with preserved ability to perform daily activities prior to injury and an ASA score of less than IV were admitted to the study. Acute OVCFs were identified on STIR-MRI or bone scintigraphy to confirm fracture acuity. Radiological follow-up for healing relied on standard lateral and AP spine radiographs to document restoration/stability of vertebral height and absence of progressive kyphosis. MRI was reserved only in case of persistent/recurrent pain to rule out ongoing bone marrow edema or complications. Pain intensity had to be at least 4 on the Visual Analogue Scale (VAS), and fractures were required to involve the thoracic or lumbar spine with classification as type A1 according to Magerl, indicating stable impaction fractures.

Exclusion criteria included any history of prior spinal surgery, the presence of local or systemic infection, malignancy with spinal involvement, and neurological deficits associated with the fracture. These criteria were established to eliminate confounding factors that could influence either treatment response or complication rates.

The final cohort included 120 patients. Demographic characteristics such as age, sex, comorbidities, and baseline functional status were recorded. Patients were retrospectively allocated to two treatment groups (vertebroplasty vs. conservative management) according to clinical judgment and patient preference. No randomization or blinding was performed. The study design was therefore retrospective and observational non-randomized, reflecting real-world clinical decision-making rather than randomized allocation. The conservative treatment group followed a standardized protocol consisting of bed rest during the acute phase, administration of non-steroidal anti-inflammatory drugs for pain control, and the use of a rigid orthopedic brace to be worn during standing and walking. The surgical group underwent percutaneous VP, performed using a minimally invasive transpedicular approach under fluoroscopic guidance. PMMA cement was injected into the vertebral body to stabilize the fracture and relieve pain. Postoperatively, patients in the VP group also used a brace for three months.

Outcome measures included the VAS for pain, the Roland–Morris Disability Questionnaire (RMDQ) for assessment of disability related to low back pain, and the Oswestry Disability Index (ODI), which is widely used in spinal surgery to evaluate overall functional impairment. These instruments were extracted from standardized clinical records collected at baseline and during routine follow-up visits approximately at 1, 3, 6, and 12 months after treatment. For patients who underwent vertebroplasty, the immediate post-procedural VAS score reported in the operative note was included when available. In addition, complications, both local and systemic, were identified through review of inpatient and outpatient records and radiology reports. Radiological evaluation with standard X-rays was performed to document fracture healing and the potential occurrence of new vertebral fractures.

### Statistical Analysis

All statistical analyses were performed using IBM SPSS Statistics, version 26.0 (IBM Corp., Armonk, NY, USA). Continuous variables were expressed as mean ± standard deviation (SD), while categorical variables were presented as absolute numbers and percentages. The normality of data distribution was assessed using the Shapiro–Wilk test. Comparisons between groups (vertebroplasty vs. conservative management) were performed using the Student’s *t*-test for independent samples for normally distributed variables and the Mann–Whitney U test for non-normally distributed data. Intragroup changes over time were analyzed with repeated-measures ANOVA or Friedman test, as appropriate. Categorical variables were compared using the Chi-square test or Fisher’s exact test. A two-tailed *p*-value < 0.05 was considered statistically significant.

## 3. Results

A total of 120 patients were included in the study. Data were collected between January 2020 and December 2022. Follow-up evaluations were conducted in person during outpatient visits, and supplementary information was retrieved from medical records when necessary. All patients met the inclusion criteria and completed the 12-month follow-up; therefore, all patients completed every scheduled follow-up visit required by the study. Sixty-three patients (21 males and 42 females; mean age 73.6 years, range 50–89) received VP, while fifty-seven (19 males and 38 females; mean age 72.7 years, range 50–89) received conservative management. All fractures were classified as type A1 according to Magerl, and all patients had osteoporosis grade I with ASA scores between II and III. Both cohorts were homogeneous in terms of demographic characteristics and baseline clinical conditions (Table 1). Group comparability reflected routine practice patterns during the study period; no random allocation was performed.

Pain and functional outcomes improved significantly in both groups over time, but with markedly different temporal patterns. Patients treated with VP experienced a rapid and substantial pain reduction immediately after the procedure, with VAS decreasing from 9.5 preoperatively to 3.9 postoperatively. Further improvement was observed during follow-up, with VAS values of 3.5 at 1 month, 2.2 at 3 months, 1.8 at 6 months, and 1.6 at 12 months. In contrast, patients in the conservative group showed a slower recovery, with VAS decreasing from 9.3 pre-treatment to 8.9 at 1 month, 7.4 at 3 months, 5.3 at 6 months, and 1.9 at 12 months.

Functional scores followed a similar trend. The ODI improved from 53.6 preoperatively to 38.8 immediately after VP, and then to 35.4 at 1 month, 21.7 at 3 months, 18.2 at 6 months, and 16.5 at 12 months. In the conservative group, ODI decreased from 52.8 pre-treatment to 49.7 at 1 month, 42.3 at 3 months, 31.8 at 6 months, and 18.7 at 12 months.

Likewise, the RMDQ scores improved from 19.9 to 12.6 postoperatively, then to 10.4, 8.7, 5.3, and 2.6 at subsequent follow-ups in the VP group, whereas in the conservative group RMDQ improved from 21.2 to 19.8, 16.7, 12.2, and 4.4, respectively.

Statistical analysis confirmed a significant intergroup difference favoring VP at 1, 3, and 6 months (*p* < 0.01), while no significant difference persisted at 12 months. Radiological healing was judged on plain radiographs; in a minority with persistent pain, an MRI was obtained, which showed resolution of bone edema when present. No symptomatic cement leakage or neurological complications were observed in the VP group, and no new adjacent-level fractures were identified during follow-up.

These results indicated that vertebroplasty offered superior short-term outcomes, providing faster pain relief and functional recovery, while long-term results converged with conservative management.

**Figure 1:**
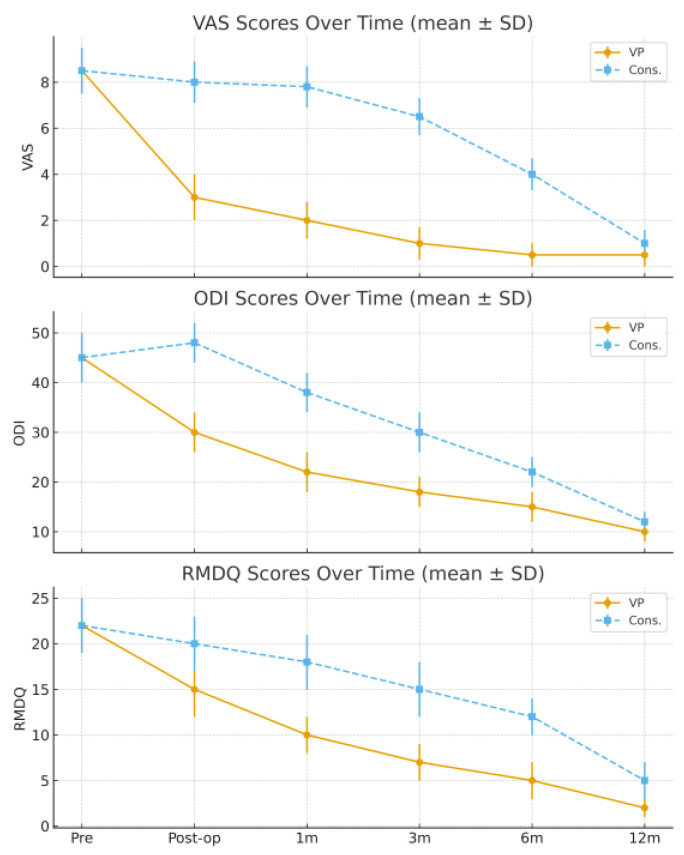
Pain intensity over time (VAS, mean ± SD). VAS (0–10), ODI (0–100), and RMDQ (0–24) values are expressed as mean ± standard deviation (SD); error bars represent SD. VP achieved a rapid decrease in VAS, ODI, and RMDQ values immediately after treatment and during the first three months, whereas improvement in the conservative group was slower and more gradual. At 12 months, both groups reached similar outcome levels.

**Figure 2:**
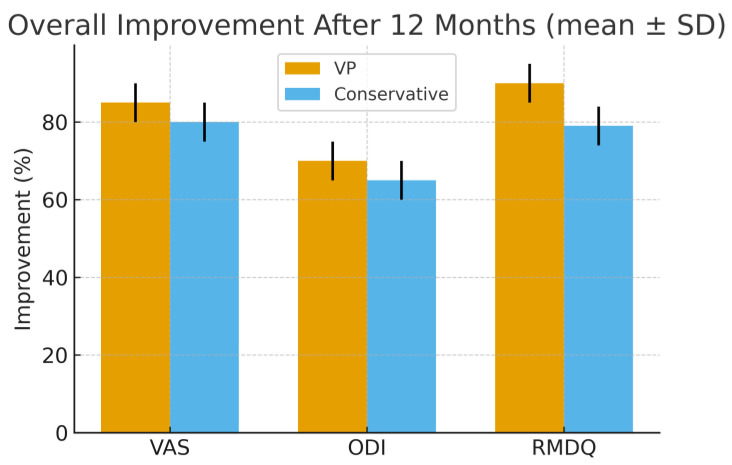
Functional outcome over time (ODI/RMDQ, mean ± SD). VAS (0–10), ODI (0–100), and RMDQ (0–24) values are expressed as mean ± standard deviation (SD); error bars represent SD. Percentage reduction of VAS, ODI, and RMDQ scores after 12 months compared with baseline values. Vertebroplasty showed greater early and overall improvement in all outcome measures, particularly within the first six months, allowing earlier functional recovery and return to daily activities.

The trends of the VAS, ODI, and RMDQ scores are summarized in Table 2 and illustrated in Figure 1 and Figure 2.

## 4. Discussion

The results of our study highlight the different temporal profiles of recovery associated with the two treatment strategies. VP provided earlier and more effective pain relief and functional recovery in the short term, whereas conservative management, although slower, ultimately reached similar levels of efficacy at one year. These findings corroborate the evidence from previous randomized controlled trials such as VERTOS and INVEST, which demonstrated the superiority of vertebral augmentation in terms of early pain relief and functional recovery [5,6,11]. Comparative studies have confirmed that the benefits of surgical intervention are particularly evident during the first six months after fracture [12,13].

Recent meta-analyses and international guidelines published between 2023 and 2025 have reinforced these observations, confirming that vertebroplasty provides faster pain relief and earlier functional improvement compared to conservative management, particularly in acute, painful fractures [14,15,16]. Nevertheless, these studies also demonstrated that functional and quality-of-life outcomes tend to converge by 12 months. Sham-controlled trials included in the most recent meta-analyses revealed partial but not complete placebo effects, supporting a true, though time-limited, therapeutic contribution of vertebral augmentation [15,16].

Selection bias remains a major concern, as patients with more severe pain, lower mobility, or reduced tolerance for prolonged immobilization were more likely to undergo vertebroplasty. This limitation is consistent with what has been reported in other observational studies and should be considered when interpreting the early differences observed between groups.

The clinical relevance of the early improvement observed in VP patients should not be underestimated. Rapid pain control allows earlier mobilization, which is crucial in elderly patients, where immobilization is often associated with complications such as muscle atrophy, pulmonary infection, venous thromboembolism, and progressive loss of independence [2,4]. The ability of vertebroplasty to provide early pain relief and mobilization remains its major advantage in the frail elderly population, where prolonged immobilization is associated with serious medical complications.

Previous epidemiological studies have shown that vertebral fractures are associated with a significant increase in morbidity and mortality, and vertebral augmentation procedures have been linked to improved survival compared with conservative management [8]. From a biomechanical perspective, cement augmentation restores part of the load-bearing capacity of the fractured vertebra, decreasing the risk of progressive deformity.

However, changes in vertebral stiffness after PMMA injection can alter load distribution along the spine, potentially predisposing adjacent levels to new fractures. Despite this theoretical risk, our results and those of several meta-analyses indicate that the overall incidence of adjacent fractures remains low, particularly when patient selection is appropriate and cement volume is carefully controlled during the procedure. The overall incidence of adjacent-level fractures does not significantly differ from conservative care when selection and technique are optimized [17]. Nevertheless, the lack of significant differences between groups at the one-year follow-up raises questions about the long-term benefit of VP. Consistent with recent analyses, between-group differences diminish by 12 months [16,18].

Some authors have reported that, although VP is associated with rapid pain reduction, the outcomes tend to converge with those of conservative treatment after 12 to 24 months [13,19]. Others have suggested that part of the observed benefit could be related to a placebo effect, as demonstrated by trials in which sham procedures showed partial efficacy in pain reduction [5]. Sham-controlled evidence indicates a genuine, though time-limited, analgesic effect of vertebroplasty [14]. Moreover, concerns about possible complications, including cement leakage, pulmonary embolism, and the development of adjacent-level fractures, remain relevant, although their incidence is generally low in experienced hands [9,12].

Another key aspect is patient selection. Evidence suggests that patients who benefit most from VP are those with acute, painful fractures showing bone edema on MRI, without significant vertebral collapse or retropulsion of bone fragments. Chronic fractures or cases with minimal pain are less likely to gain meaningful improvement. Therefore, accurate clinical and imaging evaluation is fundamental to identifying suitable candidates and avoiding overtreatment.

From an economic perspective, VP is a relatively low-cost and minimally invasive intervention, but its widespread use has to be balanced with long-term cost-effectiveness analyses. Several cost-effectiveness studies have demonstrated that both vertebroplasty and balloon kyphoplasty can be more advantageous than conservative management due to the earlier return to functional independence and the potential reduction in long-term healthcare utilization [9,11]. Comparative data between vertebroplasty and balloon kyphoplasty remain heterogeneous, with small absolute differences overall [20]. However, other authors have underlined the need for careful patient selection in order to maximize benefits and avoid unnecessary procedures [13].

Beyond clinical outcomes, the biomechanical and biological effects of vertebral cement augmentation deserve further consideration. Several studies have demonstrated that PMMA injection restores partial vertebral body strength and redistributes axial loads, thereby reducing intravertebral micromotion and nociceptive activation [6,9,11]. In addition to mechanical stabilization, the exothermic polymerization process produces a transient local hyperthermia that may influence bone remodeling at the cement–bone interface, potentially contributing to pain relief and functional recovery [9,10,11,12].

From a broader therapeutic perspective, the management of osteoporotic vertebral fractures should be regarded as a continuum rather than a single intervention. Vertebral augmentation, when indicated, should be integrated with pharmacologic treatment aimed at modifying the underlying metabolic bone disorder [1,2,17]. The initiation of antiresorptive or anabolic therapy immediately after vertebral stabilization has been associated with improved bone mineral density and a reduced risk of subsequent fractures [17,21]. Such preventive strategies are essential to interrupt the so-called “fracture cascade” that frequently follows an initial vertebral collapse [3,4].

Moreover, optimal outcomes depend on close collaboration among orthopedic surgeons, radiologists, physiatrists, and endocrinologists. Multidisciplinary care ensures early mobilization, tailored rehabilitation, and comprehensive metabolic evaluation, addressing both the mechanical and systemic determinants of fragility fractures [12,13,14]. In this context, vertebroplasty should not be interpreted as an isolated technical solution but as a component of an integrated management pathway aimed at restoring function, preserving autonomy, and preventing recurrence. Future randomized trials and updated meta-analyses published after 2023 will further refine patient selection criteria and clarify the duration of clinical benefit associated with vertebroplasty.

### Limitations of the Study

The non-randomized retrospective design represents the main limitation of this study. Although the two groups were comparable at baseline, the allocation was based on clinical judgment and patient preference, which may have introduced selection bias. Differences in pain perception, comorbidities, or willingness to undergo an invasive procedure could have influenced both treatment choice and early outcomes. Consequently, causality cannot be inferred, and the findings should be interpreted as indicative of clinical trends rather than definitive evidence of superiority.

A further limitation is the impact of the COVID-19 pandemic on patient follow-up. Due to lockdowns and restricted outpatient access during the study period, some patients were excluded or had delayed visits, which may have introduced additional bias and reduced the number of subjects with complete data [22]. These constraints are consistent with documented clinical research disruptions during the pandemic.

The relatively small sample size and the single-center design may reduce the generalizability of results. The lack of blinding could have introduced reporting bias in patient-reported outcomes, an issue already described in similar clinical trials [5,6]. Furthermore, the follow-up period of 12 months, although sufficient to assess short- and medium-term outcomes, does not allow definitive conclusions about long-term efficacy and complication rates. Larger multicenter randomized controlled trials with extended follow-up are warranted to further clarify the role of VP and establish evidence-based criteria for patient selection [10,11,21,22].

Additional limitations include the absence of an a priori power analysis, limited adjustment for potential confounders, and the single-center design, which may restrict the generalizability of our findings. A post hoc estimation nevertheless indicated adequate power for the primary outcome.

Finally, future research should also focus on combining VP with pharmacological interventions such as anabolic agents or antiresorptive therapies to improve bone healing and prevent subsequent fractures. The integration of procedural and medical management could represent the next step toward comprehensive care for patients with osteoporosis and fragility fractures.

## 5. Conclusions

Percutaneous vertebroplasty appears to provide faster pain relief and functional recovery compared with conservative management during the early follow-up period, while long-term outcomes tend to converge between the two groups.

At one year, the outcomes of the two treatment strategies converge, although VP maintains slightly better scores overall. Given its minimally invasive nature, favorable safety profile, and substantial short-term benefits, VP represents a valuable therapeutic option in selected patients with acute OVCFs. Careful patient selection remains essential, and further research is needed to consolidate evidence on its long-term efficacy and economic impact.

These results, although encouraging, should be interpreted with caution given the retrospective design and potential selection bias. Prospective randomized trials are warranted to confirm these findings.

## Figures and Tables

**Table 1:** Baseline demographic and clinical characteristics of the study population. No significant differences were observed between groups at baseline.

Variable	Vertebroplasty (n = 63)	Conservative (n = 57)	*p*-Value
Mean age	73.6 ± 8.2	72.7 ± 7.9	0.62
Female sex	42 (66.7%)	38 (66.7%)	0.99
Baseline VAS	9.5 ± 0.4	9.3 ± 0.5	0.11
Baseline ODI	53.6 ± 3.8	52.8 ± 4.1	0.37
Baseline RMDQ	19.9 ± 2.3	21.2 ± 2.6	0.14

**Table 2:** Clinical outcomes (VAS, ODI, RMDQ) in vertebroplasty and conservative groups. Mean scores of the Visual Analogue Scale (VAS), Oswestry Disability Index (ODI), and Roland–Morris Disability Questionnaire (RMDQ) over 12 months in patients treated with percutaneous vertebroplasty (VP) and conservative management (Cons.). Vertebroplasty showed faster improvement in pain and functional recovery during the first six months, while long-term results were comparable between groups.

Follow-Up	VAS VP	VAS Cons.	ODI VP	ODI Cons.	RMDQ VP	RMDQ Cons.
Pre-op	9.5	9.3	53.6	52.8	19.9	21.2
Post-op	3.9	-	38.8	-	12.6	-
1 m	3.5	8.9	35.4	49.7	10.4	19.8
3 m	2.2	7.4	21.7	42.3	8.7	16.7
6 m	1.8	5.3	18.2	31.8	5.3	12.2
12 m	1.6	1.9	16.5	18.7	2.6	4.4

## Data Availability

The data that support the findings of this study are available from the corresponding author upon reasonable request.
